# Genetic and Phenotypic Diversities in Experimental Populations of Diploid Inter-Lineage Hybrids in the Human Pathogenic *Cryptococcus*

**DOI:** 10.3390/microorganisms9081579

**Published:** 2021-07-24

**Authors:** Man You, Yuxin Monica Lin, Annamaria Dobrin, Jianping Xu

**Affiliations:** 1 Department of Biology, McMaster University, 1280 Main St West, Hamilton, ON L8S 4K1, Canada; youm1@mcmaster.ca; 2 Department of Biochemistry and Biomedical Sciences, McMaster University, 1280 Main St West, Hamilton, ON L8S 4K1, Canada; liny108@mcmaster.ca (Y.M.L.); dobrina@mcmaster.ca (A.D.)

**Keywords:** cryptococcal hybrids, mutation accumulation experiments, mitotic divisions, genome instability, phenotypic diversity, fluconazole, loss of heterozygosity

## Abstract

To better understand the potential factors contributing to genome instability and phenotypic diversity, we conducted mutation accumulation (MA) experiments for 120 days for 7 diploid cryptococcal hybrids under fluconazole (10 MA lines each) and non-fluconazole conditions (10 MA lines each). The genomic DNA content, loss of heterozygosity (LOH) rate, growth ability, and fluconazole susceptibility were determined for all 140 evolved cultures. Compared to that of their ancestral clones, the evolved clones showed: (i) genomic DNA content changes ranging from ~22% less to ~27% more, and (ii) reduced, similar, and increased phenotypic values for each tested trait, with most evolved clones displaying increased growth at 40 °C and increased fluconazole resistance. Aside from the ancestral multi-locus genotypes (MLGs) and heterozygosity patterns (MHPs), 77 unique MLGs and 70 unique MPHs were identified among the 140 evolved cultures at day 120. The average LOH rates of the MA lines in the absence and presence of fluconazole were similar at 1.27 × 10^−4^ and 1.38 × 10^−4^ LOH events per MA line per mitotic division, respectively. While LOH rates varied among MA lines from different ancestors, there was no apparent correlation between the genetic divergence of the parental haploid genomes within ancestral clones and LOH rates. Together, our results suggest that hybrids between diverse lineages of the human pathogenic *Cryptococcus* can generate significant genotypic and phenotypic diversities during asexual reproduction.

## 1. Introduction

A stable genome allows faithful transmission of genetic information from parent to progeny, ensuring genotypic and phenotypic stability within individual organisms or populations. However, genome instability is common and has been found in most organisms. Genome instability can be manifested in multiple forms and caused by various factors, such as genome doubling, chromosomal rearrangement, mitotic recombination, gene duplication, gene deletion, gene conversion, and transposition. Many of these changes involve double-strand DNA breaks, followed by repair through either the synthesis-dependent strand annealing using homologous sequences or non-homologous end joining [1,2]. Among these changes, gene deletion, gene conversion, and, to a lesser extent, mitotic recombination between homologous chromosomes, are commonly manifested as loss of heterozygosity (LOH) that can be detected using multiple molecular methods. Environmental stress, such as ultraviolet exposure and other high-energy radiation, can increase double-strand DNA breaks and elevate the LOH rates [3,4]. Spontaneous and induced LOH events were observed in diploid fungi, such as *Saccharomyces cerevisiae* and *Candida albicans* [5,6,7,8,9]. In addition, if there was a selection in favor of one of the alleles in the strain, the non-favored allele would likely be lost more frequently. For example, the antifungal drug fluconazole was shown to increase the frequencies of LOH events in *C. albicans*, resulting in aneuploidy and greater resistance to fluconazole [10]. Through LOH, diverse genotypes and phenotypes could be derived from a single diploid strain [11,12,13]. At present, most investigations of LOH focused on the impacts of environmental factors. Relatively little is known about the influences of genetic factors on LOH, such as that between genome sequence divergence of homologous chromosomes in diploid cells.

The human pathogenic *Cryptococcus* (HPC) is a group of basidiomycete yeasts and an excellent model for studying the genetic stability of hybrids and investigating how genome sequence divergence between homologous chromosomes can impact the LOH rates. HPC consists of two species complexes, the *Cryptococcus neoformans* species complex (CNSC) and the *Cryptococcus gattii* species complex (CGSC). Most strains of CNSC and CGSC are haploid, existing in one of two mating types, *MAT*α and *MAT***a**. These haploid yeast cells typically propagate asexually by budding until strains of the opposite mating-type (**a**-α) or the same mating-type (α-α) come into contact on substrates conducive for mating. The mating products are initially in a dikaryotic hyphal form that transitions into a transient diploid phase before meiosis to produce sexual spores (i.e., basidiospores). However, the diploid status can be induced and maintained through the hypha-to-yeast transition at 37 °C. In nature, most haploid strains are *MAT*α [14]. Although *MAT***a** strains are relatively rare, heterozygous diploid hybrids from **a**-α mating of HPC were frequently reported from environmental and clinical settings [6,9,10,11,12]. Both environmental and clinical hybrid isolates are either aneuploid or diploid, different from the haploid status of most non-hybrid strains [15,16,17,18,19]. Among the cryptococcal hybrids, serotype AD hybrids derived from the mating between *Cryptococcus neoformans* (serotype A) and *Cryptococcus deneoformans* (serotype D) are the most prevalent [20]. Serotype AD hybrid isolates are heterozygous at most loci but homozygous at some loci [11,15,21]. Yet, it remains unknown whether the observed homozygosity in natural hybrids was derived through meiosis, mitosis, or both.

Previous investigations of serotype AD hybrids showed that both meiosis and mitosis could generate diploid or aneuploid progeny, along with some loci being heterozygous while others being homozygous [13,15,19,21,22,23]. However, information on the genome stability of other cryptococcal hybrids, such as those of serotypes AB and BC, is very limited. In addition, two common indicators of genome instability are LOH and genomic DNA content change (i.e., ploidy change), such as the generation of aneuploidy. Aneuploidy was observed in a variety of organisms, including fungi, plants, and animals [15,24,25,26,27]. In the yeast *S. cerevisiae*, the induced chromosomal instability is often regulated by interactions between chromosomes leading to aneuploidy in cells [24]. Ploidy changes in cells are commonly observed in eukaryotes during sexual and asexual reproduction, and aneuploidy can result from disruptions in many of the steps during cell cycles [28].

Experimental evolution is a valuable tool for studying the mechanisms of adaptation to specific environmental conditions. For example, experimental evolution revealed the genetic mechanisms for fluconazole resistance and adaptations to new environments in *C. albicans*, including LOH, aneuploidy, gene duplication, and novel mutations [10,29,30]. There are multiple types of experimental evolutionary approaches. One common type is mutation accumulation (MA), where a random individual (or pair of individuals in obligate sexual species) is chosen for the transfer to establish the next generation. This process maximizes genetic drift and enables diverse types of mutations to be accumulated. In HPC, a mutation accumulation experiment involving a laboratory serotype AD strain revealed evidence of LOH and ploidy changes [13]. Researchers also found that fluconazole stress increased the LOH frequency for markers on Chromosome 1 by over 50 folds compared to non-fluconazole stress, favoring the parental homolog associated with a higher fluconazole resistance. Two Chromosome 1 genes, *ERG11* (the fluconazole target gene) and *AFR1* (the transporter for triazoles), are associated with fluconazole resistance [31,32].

In this study, we aimed to investigate the genome instability of inter-lineage hybrids of HPC, including hybrids between lineages within CGSC and hybrids between CNSC and CGSC. Specifically, we were interested in whether genomes of hybrids from evolutionarily more divergent parental strains were more stable than those from evolutionarily more similar parents. On the one hand, the increased sequence divergence between more divergent homologous chromosomes could reduce the rate of homologous recombination, resulting in more stable genomes [33]. On the other hand, having more divergent genomes in the same nucleus may make it more difficult to coordinate cellular activities during cell cycles and render the hybrid genome less stable [34]. Aside from investigating the potential impact of parental sequence divergence on hybrid genome stability, we were also interested in whether sublethal fluconazole stress would influence hybrid genome stability. To address these questions, we conducted MA experiments on seven diploid cryptococcal hybrids over 800 mitotic generations under fluconazole stress and non-fluconazole stress conditions. For all 140 MA lines, we determined their genotypes and LOH by PCR-RFLP, genomic DNA contents by flow cytometry, growth under nine conditions, and susceptibility to fluconazole. 

## 2. Materials and Methods

### 2.1. Ancestral Clones

Seven diploid *Cryptococcus* hybrids from our previous work were used as the starting clones to initiate the MA experiments [23]. These original starting clones are referred to as ancestral clones, and their descendants derived from the MA lines are called evolved clones. These hybrid ancestral clones were from seven inter-lineage crosses within CGSC and between CGSC and CNSC. They were YMD72 (progeny of B4495 × JF101, VGI × VGIII cross), YMD36 (of WM779 × JF109, VGIV × VGIII cross), YMD135 (of R265 × B4546, VGII × VGIII cross), YMD165 (of CDC15 × JF109, VNI × VGIII cross), YMD10 (of KN99a × JF101, VNI × VGIII cross), YMD88 and YMD89 (of JEC21 × B4546, VNIV × VGIII cross). The percentage of nucleotide difference based on seven single-copy nuclear genes between parental strains for each of the seven hybrids is shown in Table 1.

### 2.2. Mutation Accumulation (MA) Experiments

**Figure 1:**
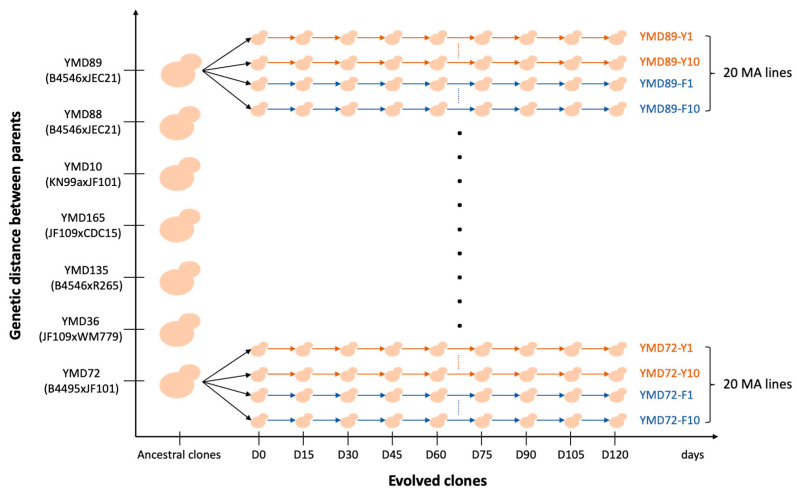
Schematic representation of mutation accumulation (MA) experiments. Seven diploid hybrids with diverse genetic backgrounds were used in this study. Each ancestral clone was used to establish 20 MA lines, including ten Y lines (cultured on YEPD agar plates) and ten F lines (cultured on YEPD + 4 µg/mL fluconazole agar plates). All MA lines were incubated at 37 °C. Each MA line underwent 40 transfers over 120 days, equivalent to about 800 mitotic generations.

It was estimated that the growth of one cell to ~10^6^ cells takes three days under our experimental conditions, representing ~20 ± 1 mitotic divisions over three days of growth [35]. In the present study, each MA line underwent 40 successive transfers at every 72 h interval, equivalent to ~800 generations in total. At each transfer, cells from one random colony were used to establish the successive MA cultures. To obtain broad patterns about the new mutations and their mutational effects, twenty MA lines were established from each ancestral clone (all from the same colony), including ten MA lines cultured on the yeast extract-peptone-dextrose (YEPD) agar plates (the Y lines) and ten MA lines maintained on YEPD + 4 µg/mL fluconazole (YEPD-FLC) agar plates (the F lines) (as illustrated in Figure 1). All MA lines were incubated at 37 °C. In every 5 transfers representing ~100 mitotic generations, an aliquot of each MA line was collected and stored at −80 °C. The MA phase lasted 120 days (i.e., ~800 mitotic generations), with the evolved cultures from days 15, 30, 45, 60, 75, 90, 105, and 120 stored and labeled as D15, D30, D45, D60, D75, D90, D105, and D120, respectively.

### 2.3. Ploidy Analysis of D120 Cultures

The genomic DNA contents of the D120 cultures of all 140 MA lines and their respective ancestral clones were determined by fluorescence-activated cell sorting (FACS), using the protocol described previously [23]. In this analysis, the haploid parental strains were used as haploid controls, and the RAS D15 strain and seven ancestral clones were used as diploid references [23,36]. Data were analyzed and visualized by ModFit LT 5.0 (Verity Software House, Topsham, ME, USA). The ploidy change of each MA line was computed as the percentage of change in peak FACS values between the D120 culture and its respective ancestral clone divided by the peak FACS value of the ancestral clone.

### 2.4. Detecting the Loss of Heterozygosity (LOH)

Thirteen nuclear genetic markers located on ten chromosomes and two mitochondrial markers were used to analyze the genotypes of the evolved clones in this study (as illustrated in Table S1). All seven ancestral clones were heterozygous at all or most of these nuclear loci [23]. Genotypic changes and occurrence of LOH among MA lines were determined using the PCR-RFLP approach. The specific markers, including their primers, PCR cycling conditions, restriction enzyme digests, gel electrophoresis, and allele scoring, were described in You and Xu [23]. The LOH rate per locus per generation was calculated as the total number of observed LOH events (total number of loci * total number of MA lines * total number of generations).

### 2.5. Growth Studies in Nine Conditions

The growth abilities of haploid parental strains, diploid ancestral clones, and the D120 cultures of all 140 MA lines were quantified in nine conditions. Briefly, fresh cells of these cultures were collected and adjusted to a final concentration of ~10^6^ cells/mL in three different liquid media, including RPMI (Roswell Park Memorial Institute; Buffalo, NY, USA) broth, YEPD broth, and YEPD-FLC broth (additional 4 µg/mL fluconazole was added to YEPD broth). These cells were inoculated in 96-well microtiter plates and incubated at three different temperatures (30 °C, 37 °C, and 40 °C) for 3 days. OD600 values were measured on day 0 and day 3. Three biological replicates of each sample under each treatment were performed with three experimental repeats.

### 2.6. Susceptibility to Fluconazole

For all D120 cultures of the 140 MA lines, the fluconazole minimal inhibitory concentration (MIC) was determined following the CLSI broth microdilution method (M27-A2) [37]. We examined fluconazole concentrations of 0 µg/mL, 0.5 µg/mL, 1 µg/mL, 2 µg/mL, 4 µg/mL, 8 µg/mL, 16 µg/mL, 32 µg/mL, 64 µg/mL, and 128 µg/mL. The MIC value was determined as the fluconazole concentration without visible growth. The MIC values of the original parental strains and the ancestral clones were also determined. One parental strain, CDC15, has a fluconazole MIC of 64 µg/mL and was used as a control [38]. The fluconazole susceptibility testing was repeated 3 times. To compare fluconazole susceptibility of the MA lines with their respective ancestral clones, we standardized the MIC values of D120 cultures of MA lines by calculating the fold changes of the evolved clones over their respective ancestral clones. The evolved clones with standardized values >2 meant that they were more than twice as resistant to fluconazole as their ancestral clones.

### 2.7. Statistical Analyses

We evaluated the genotypic diversity of the evolved clones using the Shannon-Weiner Diversity index (H). Pearson correlation tests were used to determine the relationships between the parental genetic divergence of the hybrids and LOH rates, ploidy changes, multi-locus genotypic diversity, growth rates under nine conditions, and fluconazole susceptibility of evolved clones. We examined the effects of parental genetic divergence on the observed genetic and phenotypic changes. The effects of fluconazole stress on these genetic and phenotypic changes were estimated by comparing the observed changes between F lines and Y lines. In addition, we investigated the potential impacts of ancestral clones’ fluconazole susceptibility on the changes of MICs of the evolved clones. The interaction effects of parental genetic divergence, fluconazole stress, and ancestral fluconazole susceptibility were evaluated. Generalized linear models were used to evaluate the relationships among the above factors using the R package ‘lme4’ [39]. The multi-locus genotypes (MLGs) and related statistics were determined using the R package ‘poppr’ [40]. Data visualization was done performing the R package ‘ggplot2’ [41]. All statistical analyses and visualization of data were performed using R (version 4.0.3) [42].

## 3. Results

In this study, the ploidy changes, genotypic diversity, LOH, growth rates under nine conditions, and fluconazole MIC changes of all D120 cultures were determined. The multi-locus heterozygosity patterns and LOH rates were estimated based on multi-locus genotype data. We also estimated the effects of factors (i.e., parental genetic divergence and fluconazole stress) on the observed genetic and phenotypic changes, as well as the correlations between them. Below we describe the findings.

### 3.1. Ploidy Changes among D120 Cultures of 140 MA Lines

**Figure 2:**
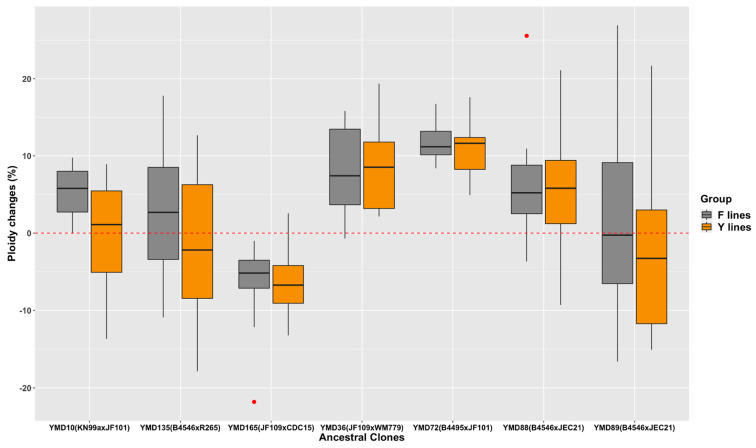
Ploidy changes of D120 cultures of MA lines from each ancestral clone. Y lines are in yellow, and F lines are in grey. Above 0 (the red dashed line) indicates an increased ploidy, and below 0 indicates a decreased ploidy.

Over 800 mitotic generations, more than 70% of D120 cultures showed different genomic DNA contents from their respective ancestral clones (as illustrated in Table S1). They had a range of changed genomic DNA contents, from ~22% less to ~27% more than their ancestral clones (as illustrated in Figure 2). Interestingly, all 20 evolved clones from YMD72 had increased genomic DNA contents. For D120 cultures from each of the remaining six hybrids, both increases and decreases were found. D120 cultures from some hybrids showed predominantly increased genomic DNA contents (e.g., 19/20 D120 cultures of YMD36), while others showed more frequently decreased genomic DNA contents (e.g., 19/20 D120 cultures of YMD165). We found that 48 out of 70 D120 cultures maintained in the presence of fluconazole (F lines) and 42 out of 70 D120 cultures maintained in the absence of fluconazole (Y lines) showed increased genomic DNA contents. Overall, the D120 cultures in the F lines had significantly higher genomic DNA contents than those in the Y lines (*p* < 0.001).

### 3.2. Genotypic Diversity among D120 Cultures of 140 MA Lines

As expected, all MA lines maintained the mtDNA genotypes as their respect ancestral clones (i.e., from the *MAT***a** parent, from the *MAT*α parent, or a recombinant genotype) at the two assayed mitochondrial loci (as illustrated in Table S1). Based on PCR-RFLP results at the 13 nuclear loci, all seven ancestral clones were heterozygous at most or all the examined nuclear loci.

Among the D120 cultures of all 140 MA lines, we observed a total of 83 multilocus genotypes (MLGs), including six ancestral MLGs and 77 *de novo* MLGs. Surprisingly, none of the D120 cultures of YMD135 had the ancestral MLG (MLG.29). In addition, the D120 cultures from the seven ancestral clones showed different genotypic diversities (as illustrated in Table 2). Specifically, the D120 cultures of YMD89 had the highest genotypic richness with 17 MLGs, followed by that of YMD88 with 16 MLGs. In contrast, the D120 cultures of YMD165 had the lowest richness with only two MLGs. Consequently, the D120 cultures of YMD89 had the greatest genotypic diversity (H = 2.76), whereas the D120 cultures of YMD165 showed the lowest genotypic diversity (H = 0.42).

According to their multilocus heterozygosity patterns (MHPs), these seven ancestral clones were assigned to two MHPs, MHP.16 and MHP.74 (as illustrated in Table S1). Among the 140 D120 evolved cultures, 15 from the Y lines and 11 from the F lines shared MHP.74, and three each from the Y lines and F lines shared MHP.16. None of them reverted to the original *MAT***a** or *MAT*α haploid parental genotypes (i.e., MHP.1 and MHP.19). Among the remaining 108 MA lines, we found a total of 70 *de novo* MHPs, including eight MHPs shared between Y lines and F lines (MHP.35, MHP.39, MHP.50, MLP.69, MHP.70, MHP.71, MHP.72, and MHP.73), 34 MHPs were only found among Y lines, and 28 unique MHPs were found only in F lines.

### 3.3. LOH among 140 MA Lines

Among the 13 nuclear loci, LOH events were found at 11 loci. The only two loci without any detected LOH were the *MAT* locus and *CGNA* (as illustrated in Table S1). Among the 11 loci with LOH, there were notable variations in the LOH frequencies. For example, locus *CNI01350* had the highest LOH frequency with 3.3 × 10^−4^ LOH events per MA line per mitotic division, while locus *ERG11* had the lowest LOH frequency with 5.36 × 10^−5^ LOH events per MA line per mitotic division. Aside from the overall differences in LOH frequencies among loci, there were also differences in the timing of the LOH events (as illustrated in Table S2). For example, some LOH events occurred very early during MA (e.g., within 100 mitotic generations), while others happened much later (e.g., after 700 mitotic generations). 

In total, we detected 213 LOH events (97 in Y lines and 116 in F lines) accumulated over 800 mitotic generations among the 140 MA lines, corresponding to 1.27 × 10^−4^ LOH events per sample per locus per mitotic division. Of the two MA conditions, Y lines had a LOH frequency of 1.15 × 10^−4^ LOH events per MA line per mitotic division, whereas F lines had a frequency of 1.38 × 10^−4^ LOH events per MA line per mitotic division. The LOH rates varied among MA lines derived from different ancestors (as illustrated in Table S2). The lowest was found from Y lines of YMD165 with a LOH rate of 8.33 × 10^−6^ LOH events per MA line per mitotic division. The highest was found from F lines of YMD88 with a LOH rate of 2.92 × 10^−4^ LOH events per MA line per mitotic division. In general, we found that Y lines and F lines derived from the same ancestral clones had similar LOH rates. However, in YMD88, the LOH rate of F lines was two times more than that of Y lines.

### 3.4. Growth Rates of D120 Cultures under Nine Environmental Conditions

**Figure 3:**
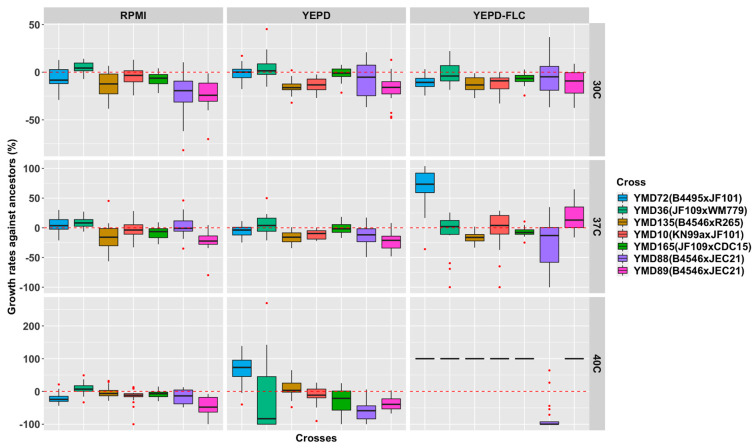
Growth rates of D120 cultures of 140 MA lines derived from each ancestral clone under 9 environmental conditions.

The D120 cultures showed diverse growth rates under the nine tested environmental conditions (as illustrated in Figure 3). Overall, the growth rates of D120 cultures were lower than their respective ancestral clones at 30 °C and 37 °C but higher at 40 °C. The results indicate that the cultures evolved at 37 °C had superior growth at 40 °C than their respective ancestral clones. We also observed growth differences among the three media. On average, the growth rates of D120 cultures were lower than their ancestral clones at all three temperatures in the RPMI and YEPD media. However, the D120 cultures grew faster than their respective ancestral clones in the YEPD-FLC medium at 37 °C and 40 °C. The average growth rates of D120 cultures from both Y lines and F lines were significantly greater at 40 °C than at 37 °C in the YEPD-FLC medium (*p* values < 0.001). The only exceptions were D120 cultures from the ancestral clone of YMD88 showing decreased growth in the YEPD-FLC medium at 40 °C. The findings suggest that the MA temperature at 37 °C can increase the growth ability of the evolved clones at a higher temperature (i.e., 40 °C).

### 3.5. Fluconazole MIC Changes of D120 Cultures

**Figure 4:**
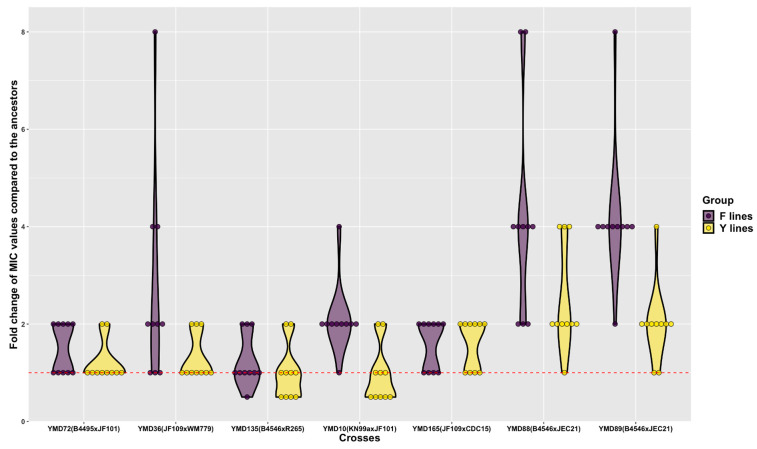
Fold changes of fluconazole MIC values among D120 cultures of 140 MA lines derived from each ancestral clone.

Among the D120 cultures, the fold changes of MIC values from their respective ancestral clones ranged from 0.5 to 8 (as illustrated in Table S1 and Figure 4). Of the 140 D120 cultures, 82 (~59%) showed an increased fluconazole MIC, including four from F lines having an 8-fold increase, 16 from F lines, and 4 from Y lines having a 4-fold increase, and 30 from F lines and 28 from Y lines having a 2-fold increase. In total, 50 out of 70 (~71%) D120 cultures from F lines and 32 out of 70 (~46%) D120 cultures from Y lines had higher MIC values than their respective ancestor clones. In contrast, D120 cultures from one F line and nine Y lines had decreased fluconazole MIC compared to that of their ancestral clones. The remaining 48 (29 from Y lines and 19 from F lines) maintained the same MIC values as their respective ancestors. Overall, after 800 mitotic divisions, 130 out of the 140 MA lines (~93%) maintained or increased their fluconazole MIC.

### 3.6. Relationship between Parental Genetic Divergence and the Genetic and Phenotypic Changes among MA Lines

**Figure 5:**
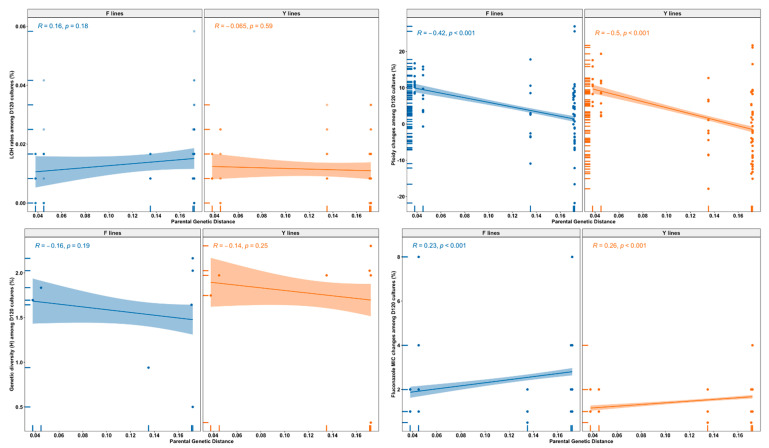
Relationships between parental genetic distance and genetic and fluconazole MIC changes of D120 cultures of 140 MA line. Genetic changes of evolved clones include LOH rates, ploidy changes, and genotypic diversity.

**Figure 6:**
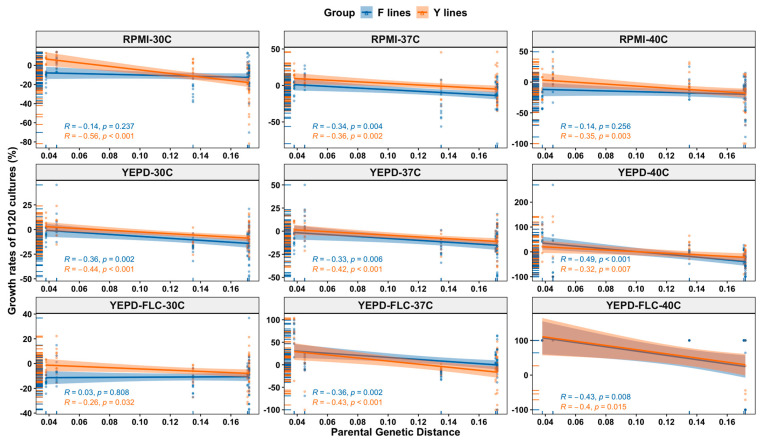
Relationships between parental genetic distance and growth rates of D120 cultures of 140 MA lines under 9 environmental conditions.

The genetic distance between parental genomes within hybrids showed several significant correlations with genetic and phenotypic changes of evolved hybrid MA clones (as illustrated in Figure 5). Specifically, parental genetic distance within hybrids was significantly positively correlated with fluconazole MIC changes of both the Y lines (r = 0.26, *p* < 0.001) and the F lines (r = 0.23, *p* < 0.001). In contrast, it was significantly negatively correlated with ploidy changes of the Y lines (r = −0.5, *p* < 0.001) and the F lines (r = −0.42, *p* < 0.001). We also observed negative correlations between parental genetic distance and growth rates of the Y lines under all nine conditions and those of the F lines under six conditions (as illustrated in Figure 6). For example, there were negative correlations in YEPD-FLC medium at 40 °C (r = −0.4, *p* = 0.015 for Y lines; r = −0.43, *p* = 0.008 for F lines). Overall, our data indicate that parental genetic divergence significantly affected ploidy changes, fluconazole MIC changes, and growth rates under most tested conditions of both the Y lines and the F lines, as well as LOH rates of the F lines.

### 3.7. Impacts of Fluconazole Stress on Genetic and Phenotypic Changes among MA Lines

Pairwise comparisons of D120 cultures between Y lines and F lines were used to evaluate the potential effects of fluconazole stress on the observed genetic and phenotypic changes. Overall, we found that D120 cultures from the Y lines and the F lines showed similar genetic diversity. There was no statistically significant difference in LOH rates between F lines and Y lines. Interestingly, D120 cultures from F lines showed greater increases in ploidy and fluconazole MIC than those from Y lines (*p* values < 0.001). In contrast, D120 cultures from the Y lines had on average higher growth rates than those from F lines under 7 of the 9 conditions, except for the YEPD-FLC medium at 37 °C and 40 °C. These results indicate that fluconazole stress could affect the ploidy levels and fluconazole MIC values of the evolved clones during mitotic divisions.

### 3.8. Relationships between the Observed Genetic Changes and Phenotypic Changes among MA Lines

**Figure 7:**
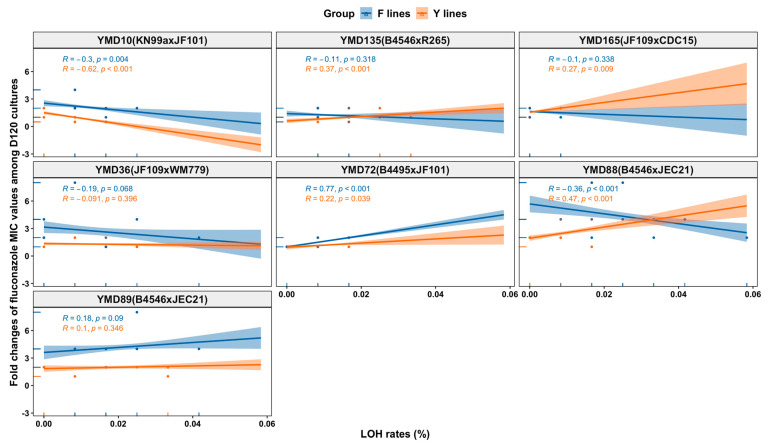
Relationship between LOH rates and fluconazole MIC value changes of D120 cultures of 140 MA lines from each ancestral clone.

We found several statistically significant correlations between the observed genetic changes (i.e., ploidy changes and LOH rates) and phenotypic changes (i.e., fluconazole MIC changes and growth rates) among the D120 cultures. For example, LOH rates of F lines were significantly positively correlated with fluconazole MIC changes (r = 0.29, *p* = 0.013), while negatively correlated with growth rates at 40 °C in the YEPD-FLC medium (r = −0.61, *p* < 0.001). However, for both F lines and Y lines, diverse correlations were observed when the analyses were conducted for the lines derived from individual strains (as illustrated in Figure 7). For example, LOH rates were significantly negatively correlated with fluconazole MIC changes of Y lines from YMD10 (r = −0.62, *p* < 0.001), while positively correlated with those of Y lines from YMD135 (r = 0.37, *p* < 0.001). Interestingly, for MA lines derived from YMD88, LOH rates were significantly positively correlated with fluconazole MIC changes of Y lines (r = 0.47, *p* < 0.001) but negatively correlated with those of F lines (r = −0.36, *p* < 0.001). We found similarly diverse correlations between LOH rates and growth rates, with ancestral clones, MA conditions (i.e., fluconazole stress and non-fluconazole condition), and growth conditions contributing to the positive and negative correlations. For example, the D120 cultures of the Y lines from YMD165 showed a statistically significant positive correlation between their LOH rates and growth rates in the YEPD-FLC medium at 37 °C (r = 0.71, *p* = 0.022; as illustrated in Figure S1).

**Figure 8:**
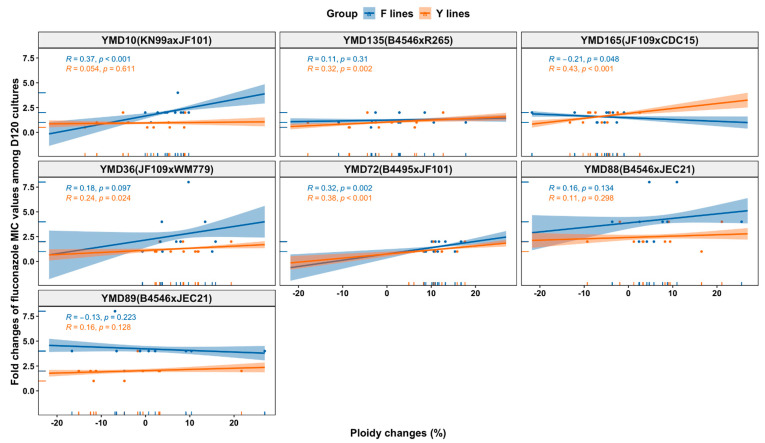
Relationship between ploidy changes and fluconazole MIC value changes of D120 cultures of 140 MA lines from each ancestral clone.

Similar to the observed relationships between LOH rates and phenotypic changes among MA lines, a diversity of relationships between ploidy changes and phenotypic changes were also found. For example, we observed overall positive correlations between ploidy changes and growth rates for D120 cultures in Y lines in the RPMI medium (at 37 °C, r = 0.34, *p* = 0.005; and at 40 °C, r = 0.31, *p* = 0.009) but not in F lines. The D120 cultures from both F lines and Y lines from some ancestral clones showed positive correlations between ploidy changes and fluconazole MIC changes and growth rates, while those of others were negatively correlated or showed no association (as illustrated in Figure 8 and Figure S2). For example, ploidy changes were positively correlated with fluconazole MIC changes of Y lines (r = 0.43, *p* < 0.001) but negatively associated with that of F lines (r = −0.21, *p* = 0.048). For growth rates in different media, ploidy changes were only negatively associated with the growth rates of D120 cultures in Y lines from YMD72 in the YEPD medium at 30 °C (r = −0.69, *p* = 0.027) and of Y lines from YMD89 in the YEPD-FLC medium at 30 °C (r = −0.7, *p* = 0.025). Overall, the relationships between the observed genetic changes and phenotypic changes varied among Y lines and F lines from different ancestral clones.

## 4. Discussion

Our study used mutation accumulation experiments to investigate the genome stability of seven diploid cryptococcal hybrids during mitotic divisions. We evaluated the effects of parental genetic divergence and fluconazole stress on genome stability, growth ability, and fluconazole susceptibility, as well as relationships between genetic and phenotypic changes. Below we discuss the potential mechanisms and implications for our findings.

### 4.1. Contributions of Parental Divergence to Genetic Changes

We investigated the effects of parental genetic divergence on LOH rates, ploidy changes, and genotypic diversity. For both Y lines and F lines, parental genetic divergence showed significant impacts on ploidy changes while not on LOH rates or genetic diversity. Our findings suggest that MA lines derived from more divergent parental pairs were like to have more stable ploidy levels over generations. 

Cryptococcal lineages show from 2–17% of nucleotide sequence divergence at the whole genome level and are believed to have diverged from each other for 4.7 to 100 million years [43,44,45,46,47,48,49]. Previous studies revealed genetic incompatibilities between divergent parental strains and structural differences in chromosomes among divergent lineages in the human pathogenic *Cryptococcus* [50,51,52,53]. Ancestral clones (i.e., original hybrids), derived from genetically more closely related haploid parental strains, contained more similar chromosomal homologs with higher sequence similarities than that from more distantly related haploid parental strains. The more similar sequences and chromosomal structures between homologs of hybrids derived from more closely related haploid parents could contribute to a higher rate of homologous recombination and ploidy change during the extended asexual reproduction. Indeed, previous studies showed that as the genetic divergence between homologous sequences increases, the rate of homologous recombination decreases [54,55].

### 4.2. Contributions of Fluconazole Stress to Genetic Changes

By comparing the observed genetic changes between Y lines and F lines, we found that the tested fluconazole stress (4 µg/mL) contributed significantly to the ploidy status of MA lines. The evolved clones under fluconazole stress (F lines) had higher ploidies on average than those under non-fluconazole stress lines (Y lines). Evidence that fluconazole stress can induce ploidy changes (e.g., aneuploidy) was reported in many species, such as *Candida albicans* and *Cryptococcus* *neoformans* [13,56,57]. Aneuploidy is generated due to the loss or gain of chromosomes or chromosomal segments within a genome. In humans, aneuploidy is often detrimental and linked to a variety of genetic disorders, such as Down syndrome (trisomy 21). However, when cells are exposed to stress, aneuploidy can provide a quick response through changes in gene dosage [26]. Fluconazole is an antifungal drug commonly administered to treat cryptococcosis, a severe disease caused by strains of HPC. Fluconazole can induce aneuploidy by disrupting the normal synchronization of chromosomal and cytoplasmic divisions in *C. albicans* [58]. In *C. neoformans*, pleiotropic effects of fluconazole on cell growth and mitotic division likely contribute to select for increased copies of chromosomes or chromosomal segments containing genes related to fluconazole stress response [56]. The continued fluconazole pressure during mutation accumulation for the F lines selects for cells with maintained or increased fluconazole resistance, contributing to their increased prevalence in subsequent MA cultures.

### 4.3. Interaction Effects of Factors on the Observed Changes among MA Lines

In this study, we focused on the impacts of two factors separately on the genotypic and phenotypic changes of these hybrids during mutation accumulation. The results demonstrated that: (i) parental genetic distance can influence ploidy changes and growth rates among the D120 cultures of the MA lines under several conditions, and (ii) fluconazole stress can impact ploidy changes and fluconazole MIC changes. Interestingly, the analyses also indicated interaction effects of these two factors, with certain correlations found only in one of the two MA conditions. Indeed, our analyses showed interactions between parental genetic divergence and MA conditions on most observed phenotypic and genetic changes among the D120 cultures, including LOH rates, ploidy changes, genetic diversity, fluconazole MIC changes, and growth rates at 30 °C and 37 °C in the RPMI medium and at 40 °C in the YEPD-FLC medium. Because natural environments are heterogenous and cryptococcal hybrids can disperse across geographic regions and ecological niches, divergent genotypes with different phenotypic properties could be evolved and selected from single hybrids through sexual reproduction, which could bring negative impacts to patients and our healthcare system.

### 4.4. Relationships between Ploidy Changes and Phenotypic Changes

Aneuploidy refers to gains and/or losses of individual chromosome (s) or chromosomal segment (s) from the normal chromosome set. The ploidy changes in aneuploid strains could directly alter the transcription level of many genes on the affected chromosome (s) and indirectly change the expressions of genes on other chromosomes. This is because most genetic and metabolic pathways contain genes from multiple chromosomes, and when some components of a pathway in one chromosome are activated or inhibited, other components in other chromosomes could also be affected. In the present study, we found that most MA lines deviated from the genomic DNA contents of their ancestral clones after ~800 mitotic generations, with both increased and decreased ploidy levels compared to their ancestral clones (as illustrated in Table S1). Furthermore, ploidy changes were significantly correlated with fluconazole MIC changes and growth rates under several conditions. Previous research showed that specific aneuploidies can impact phenotypes in human pathogenic yeasts. For example, increased ploidies in several yeasts have shown enhanced antifungal drug resistance and greater growth rates under specific conditions [59,60,61]. However, decreased copy numbers of specific chromosomes could also lead to increased drug resistance. For example, in *C. albicans*, monosomy of chromosome 5 increases resistance to several antifungal drugs [62]. While specific ploidy changes can enhance the strains’ survival under the selected stress conditions, in the absence of such stress, aneuploidy can have detrimental effects. Moreover, if there is no selective pressure to maintain the aneuploidy, aneuploidy often returns to euploidy [63]. At present, the conditions under which aneuploidy of the MA lines in our study will return to euploidy remain to be investigated.

### 4.5. Relationships between LOH and Phenotypic Changes

While no correlation was found between LOH rates and growth rates under most of the tested conditions for both Y lines and F lines, significant correlations were found for MA lines derived from specific ancestral clones under certain conditions (as illustrated in Figure S1). In addition, we found notable influences of LOH rates on fluconazole MIC changes of both Y lines and F lines (as illustrated in Figure 7). 

Two genes on chromosome 1 were confirmed to be associated with fluconazole resistance in *C. neoformans*, *ERG11* and *CnAFR1*. *ERG11* encodes the fluconazole target enzyme lanosterol 14-α-demethylase, and *CnAFR1* is an ATP binding cassette (ABC) transporter-encoding gene [31,32,38]. A previous MA study on a serotype AD hybrid found that in the presence of high fluconazole concentration (32 µg/mL), most LOH events happened on chromosome 1, resulting in the loss of fluconazole-susceptible allele of *ERG11* [13]. Differently, in this study, we only observed six LOH events on the *ERG11* locus among D120 cultures of 140 MA lines, including three in Y lines and three in F lines. The reason for the different LOH rates of *ERG11* between these two studies was likely due to the different fluconazole concentrations used. In the current study, we used 4 µg/mL of fluconazole (vs. 32 µg/mL in the previous study), and the parental haploid strains could all grow on the agar medium containing 4 µg/mL of fluconazole. Another study found that chromosome 4 is the second most frequently formed disomy at high concentrations of fluconazole [64].

In this study, we did not find any relationship between LOH at any specific locus/loci and the increased fluconazole MIC values. However, by comparing heterozygous rates of the examined 13 nuclear loci together to fluconazole MIC changes, we found a significantly negative correlation between heterozygous rates and fluconazole MIC changes (r = −0.28, *p* < 0.001) among D120 cultures of F lines (r = 0.28, *p* < 0.001). In contrast, no correlation was found between them among the Y lines. In addition, the nuclear locus *CNI01350* had the highest LOH rate, and it is a QTL (quantitative trait locus) associated with cell size in *Cryptococcus neoformans* [38]. Altogether, our data suggest that the 4 µg/mL fluconazole stress in this study had an overall effect on heterozygosity but was not sufficient as a selective force for LOH at any specific locus.

## 5. Conclusions

In this study, we investigated the genetic and phenotypic changes of cryptococcal diploid hybrids after ~800 mitotic generations and examined the effects of potential factors on the observed changes. Our analyses revealed that asexual reproduction of these hybrids under laboratory conditions could generate high genotypic and phenotypic diversities. Overall, we found that neither the parental genetic divergence of each hybrid ancestral clone nor the mutation accumulation condition (i.e., fluconazole stress and non-fluconazole condition) could be used to predict all the observed variations. Instead, both the parental genetic divergence and the MA conditions, as well as their interactions, contributed to the observed genotypic and phenotypic changes among the MA lines. Interestingly, significant correlations among MA lines from individual ancestors were frequently observed between certain genetic changes and phenotypic changes. In addition, most evolved lines showed increased fluconazole resistance, especially those maintained on medium containing fluconazole, suggesting that the treatment of infection by cryptococcal hybrids could present additional challenges over those by non-hybrids. For example, the presence of sublethal dose antifungals for the treatment of infections by hybrids might not kill these pathogens but instead facilitate the evolution of diverse genotypes and increased drug resistance. Together, our findings indicate the enormous potential of cryptococcal hybrids to generate genotypic and phenotypic diversities and that multiple factors can impact the production of such diversities. Future analyses of the genomes and transcriptomes of the evolved clones could help reveal their detailed genetic changes and the potential mechanisms contributing to the observed phenotypic differences among these clones.

## Figures and Tables

**Table 1:** Information on ancestral clones used in this study.

Crosses		Ancestral Clones	*MAT*a Parent (Lineage)	*MAT*α Parent (Lineage)	Parental Haploid Genome Divergence
Inter-lineage crosses within CGSC	VGI × VGIII	YMD72	B4495 (VGI)	JF101 (VGIII)	0.038
	VGIV × VGIII	YMD36	JF109 (VGIII)	WM779 (VGIV)	0.045
	VGII × VGIII	YMD135	B4546 (VGIII)	R265 (VGII)	0.135
Inter-lineage crosses between CNSC and CGSC	VNI × VGIII	YMD165	JF109 (VGIII)	CDC15 (VNI)	0.171
		YMD10	KN99a (VNI)	JF101 (VGIII)	0.171
	VNIV × VGIII	YMD88	B4546 (VGIII)	JEC21 (VNIV)	0.172
		YMD89	B4546 (VGIII)	JEC21 (VNIV)	0.172

**Table 2:** Genetic diversity among MA lines of each cross.

Ancestral Clones	Number of MA Lines	Number of Multi-Locus Genotypes (MLGs)	Shannon-Weiner Diversity Index (H)
YMD72	20	9	2.06
YMD36	20	14	2.39
YMD135	20	11	2.00
YMD165	20	2	0.42
YMD10	20	14	2.53
YMD88	20	16	2.69
YMD89	20	17	2.76

## Data Availability

We confirm that all data supporting the reported results are provided in this article, including in the Supplementary Materials.

## Supplementary Materials

The following are available online at https://www.mdpi.com/article/10.3390/microorganisms9081579/s1, Figure S1: Relationships between LOH rates and average growth rates of D120 cultures of 140 MA lines derived from each ancestor under each environmental condition; Figure S2: Relationships between ploidy changes and average growth rates of D120 cultures of 140 MA lines derived from each ancestor under each environmental condition; Table S1: Details of MA lines, ploidy changes, fluconazole MIC changes, multi-locus genotypes, multi-locus heterozygosity patterns, nuclear genotypes, and mitochondria genotypes, and Table S2: The occurrence of LOH events among MA lines during mitotic divisions.
