# Supplementary material for: Genetic and Phenotypic Diversities in Experimental Populations of Diploid Inter-Lineage Hybrids in the Human Pathogenic Cryptococcus
Source: Microorganisms. 2021 Jul 24;9(8):1579. doi: 10.3390/microorganisms9081579 (PMC8398696; doi:10.3390/microorganisms9081579)
Supplement: Supplementary file 1 [file microorganisms-09-01579-s001.zip › microorganisms-1283959-supplementary.pdf]

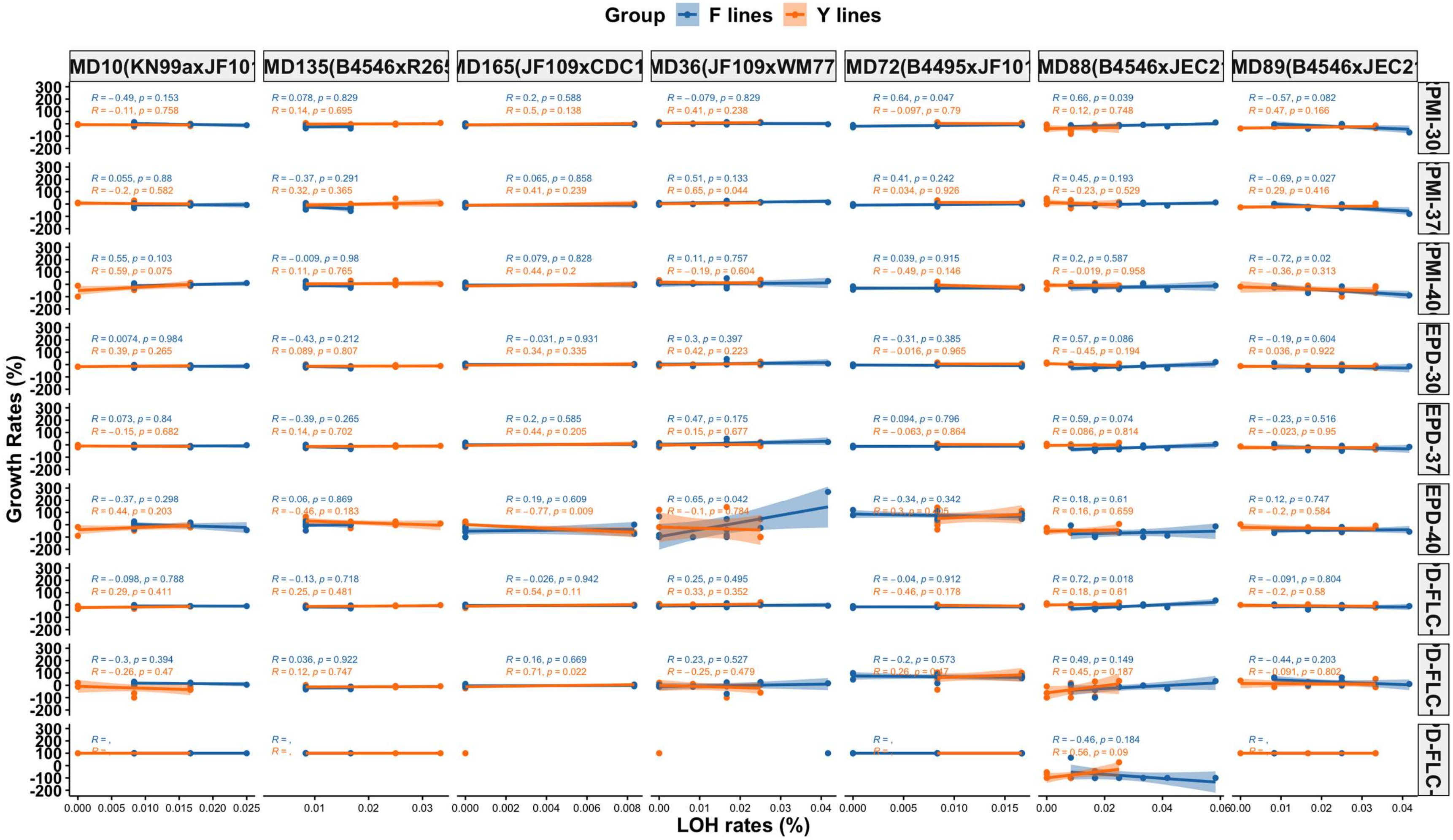

Figure S1. Relationships between LOH rates and average growth rates of D120 cultures of 140 MA lines derived from each ancestor under each environmental condition.

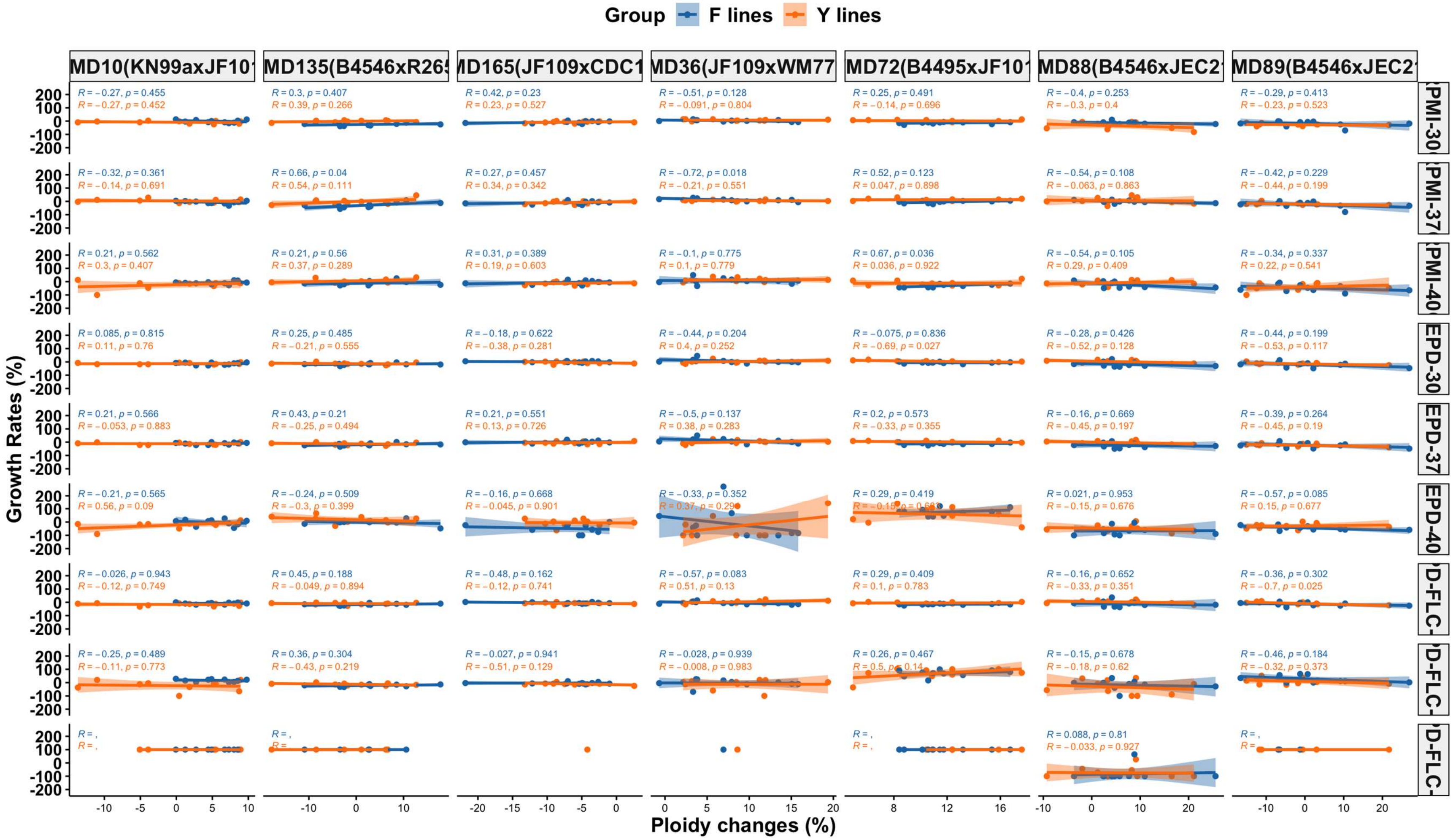

Figure S2. Relationships between ploidy changes and average growth rates of D120 cultures of 140 MA lines derived from each ancestor under each environmental condition.
